# Rust HUBB: DNA barcode-based identification of Pucciniales

**DOI:** 10.1186/s43008-023-00132-7

**Published:** 2024-02-25

**Authors:** Patricia Kaishian, Christopher R. K. Layug, Mark Anderson, Diane R. Berg, M. Catherine Aime

**Affiliations:** 1 Department of Botany and Plant Pathology, Purdue University, 915 W. State Street, West Lafayette, IN 47907 USA; 2 New York State Museum, 3140 Cultural Education Center, Albany, NY 12230 USA

**Keywords:** *Puccinia*, *Coleosporium*, *Uromyces*, *Phakopsora*, *Ravenelia*, Uredinales

## Abstract

Rust fungi (Pucciniales, Basidiomycota) are a species-rich (ca. 8000 species), globally distributed order of obligate plant pathogens. Rust species are host-specific, and as a group they cause disease on many of our most economically and/or ecologically significant plants. As such, the ability to accurately and rapidly identify these fungi is of particular interest to mycologists, botanists, agricultural scientists, farmers, quarantine officials, and associated stakeholders. However, the complexities of the rust life cycle, which may include production of up to five different spore types and alternation between two unrelated host species, have made standard identifications, especially of less-documented spore states or alternate hosts, extremely difficult. The Arthur Fungarium (PUR) at Purdue University is home to one of the most comprehensive collections of rust fungi in the world. Using material vouchered in PUR supplemented with fresh collections we generated DNA barcodes of the 28S ribosomal repeat from > 3700 rust fungal specimens. Barcoded material spans 120 genera and > 1100 species, most represented by several replicate sequences. Barcodes and associated metadata are hosted in a publicly accessible, BLAST searchable database called Rust HUBB (Herbarium-based Universal Barcode Blast) and will be continuously updated.

## INTRODUCTION

Rust fungi (Pucciniales, Basidiomycota) are the largest, in terms of species, group of plant pathogenic fungi. Pucciniales contains ca. 8000 described species in 7 suborders, 18 families, and ca. 140 genera (Cummins & Hiratsuka 2003; Kirk et al. 2008; Aime and McTaggart 2021) that makes up ca. 20% of described Basidiomycota. Rust fungi are globally distributed and infect a diverse range of vascular plant hosts. Diseases caused by rusts affect numerous agriculturally significant plants such as wheat (*Triticum* spp.), soybean (*Glycine max*), corn (*Zea mays*), coffee (*Coffea* spp.), and apples (*Malus domestica*), to list a few, making them of serious concern for global food security (Rossman 2008). Rust fungi are also primary agents of invasive diseases and have the potential to drive host extinctions and change ecological landscapes (Helfer 2014; Berthon et al. 2018). Research into biological mechanisms to control rust diseases is an active and promising field (Gomez-Zapata 2022). Alternatively, rusts are also studied for their capacity as biocontrol agents themselves against invasive plants (Evans 1993; Bruckart III et al. 2010).

Rust fungal species are challenging to identify. As obligate phytopathogens, rust fungi have evolved five different spore stages and alternation between two unrelated host plants to complete their life cycle. Traditional identification is based on spore and sorus morphology as well as host identification (e.g., Petersen 1974; Ramsbottom 1912; Sato and Sato 1985); however incomplete data on spore stages and alternate hosts for many rust species can lead to mis-identifications or even the inability to make an identification based on current host range data. Additionally, convergent evolution within different spore stages further complicates accurate identification (Aime and McTaggart 2021), as does the fact that only the telial spore stage, which is typically only produced once a year at the end of the life cycle, provides enough characters to be of much diagnostic value at the species rank.

DNA barcoding (Hebert et al. 2003) is an identification method that involves selecting a specific DNA locus which is shared across the target taxa. The ideal locus varies between species, with little variation within species. Sequences from unknowns are then compared to a database of sequences from known sources to derive a “match” or identification (Ratnasingham and Herbert 2007, 2013). Within fungi, there have been three regions of the ribosomal repeat utilized most frequently in barcoding: 1) the large subunit (LSU which is also referred to as 26S or 28S); 2) the small subunit (SSU, or 18S); and, 3) the internal transcribed spacer region (ITS, comprising three sections: ITS1, the 5.8S, and ITS2) (Seifert 2009). The ITS region is the universal barcode for fungi (Schoch et al. 2012). However, intragenomic variation within the ITS region appears widespread in Pucciniales, making this region unreliable as a barcode in this group (e.g., McTaggart & Aime 2018; Bradshaw et al. 2023). In contrast, the 28S region has been widely adopted for identification of rust fungi (Abbasi et al. 2023; Harvey et al. 2023; Huaman-Pilco et al. 2023; Kaishian et al. 2023).).

In this paper we publish a new online public database, Rust HUBB (Herbarium-based Universal Barcode Blast), that contains verified, vouchered 28S sequence barcodes for > 3700 rust fungal specimens. Barcoded material spans 120 genera and > 1100 species on all continents. Rust HUBB will be continually updated with new, vouchered and verified sequence data and welcomes contributions from the community.

## MATERIALS AND METHODS

**Fig. 1:**
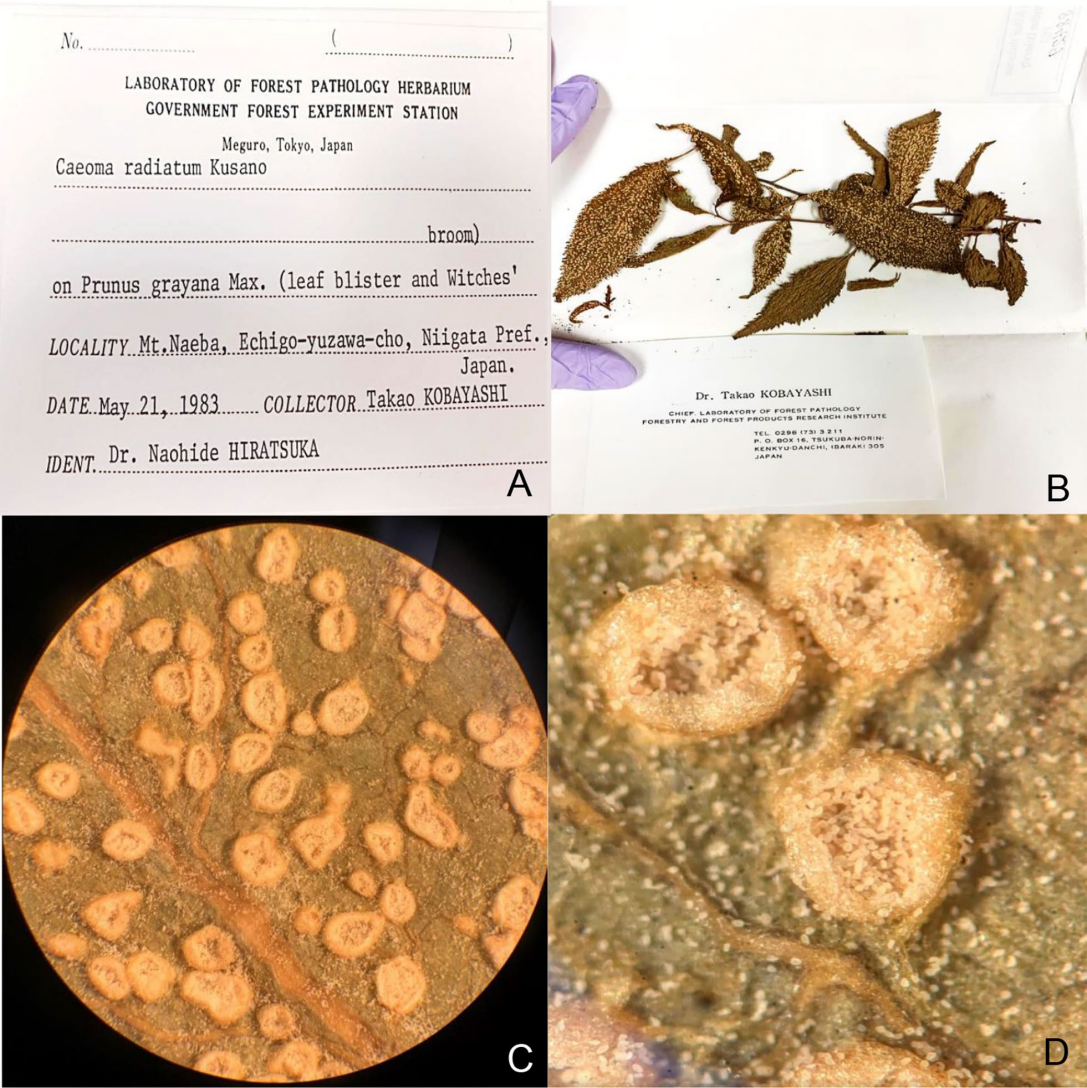
The Arthur Fungarium is home to one of the largest collections of rust fungi in the world. Pictured is one such specimen*, Ceoma radiatum* on *Prunus grayana*, collected from Mt. Naeba, Echigo-yuzawa-cho, Niigata Prefecture, Japan in 1983. The specimen was collected by Dr. Takao Kobayashi and sent to the Arthur Fungarium from the Laboratory of Forest Pathology Herbarium in Japan. **A**–**B**) Aecia of *C. radiatum* under dissecting microscope; **C** Specimen taken from collection for excision and DNA extraction; **D** original specimen label

Rust fungal specimens preserved in the Arthur Fungarium (PUR) at Purdue University, West Lafayette, IN, USA, were utilized in this work (Fig. 1). Included were specimens from every continent except Antarctica. While specimens collected after 1980 were prioritized due to the increased likelihood of harboring intact DNA, specimens as old as 1895 were included. Starting with samples dated from 1980 onwards, we selected up to 3 specimens, ideally collected from different regions or localities, representing each species (with some replicate sequencing of the same isolate to test methods). This sampling was supplemented with older specimens and/or material vouchered in other herbaria representing genera or species that would give the broadest representation across the rust tree of life.

**Fig. 2:**
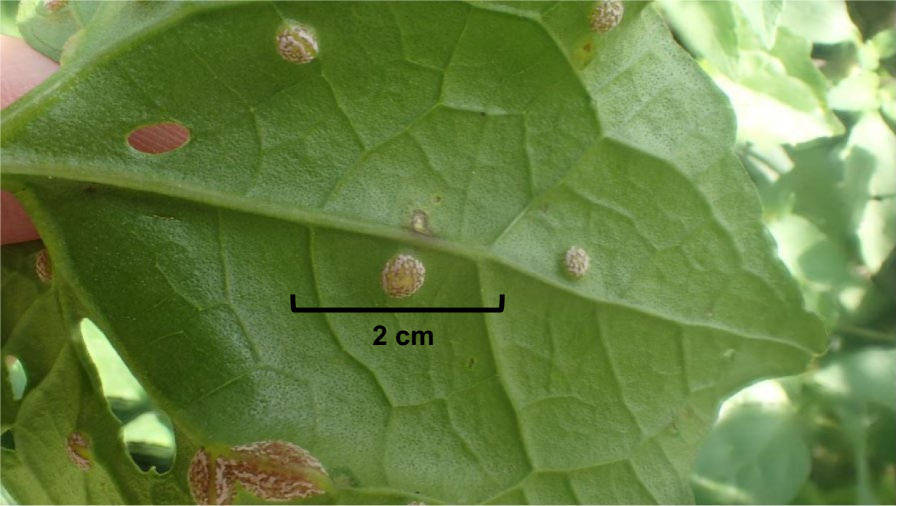
Telial sori of *Puccinia spegazzinii* on a leaf of *Mikania* sp. This leaf contains numerous sori suitable for excision. Following our procedural guidelines, about ¼ of the sorus in the center of the leaf could be excised with a razor blade, and then crushed and used for extraction

Small portions (typically about 0.5 cm^2^) of rust fungal sori and infected plant material were excised under an Olympus microscope at 50 × and carefully removed from the surrounding plant tissue, thus maximizing the proportion of fungal DNA within an excision (Fig. 2). In order to reduce any likelihood of cross-contamination between species, the following methods were developed for each excision. The microscope stage and lab bench were cleaned before use. Gloves were worn when handling specimens and were wiped thoroughly between specimens. A clean piece of paper was placed on the dissecting scope stage for each rust specimen. Each specimen was cut and crushed finely on clean paper with a newly opened razor blade, then carefully scraped from paper with the blade and transferred into Omega Bio-Tek disruptor tubes. DNA was extracted from these samples using the E.Z.N.A. Plant DNA DS kit (manufacturer), following the manufacturer’s protocols, except that incubation in protein K overnight as opposed to 30 min and the elution volume was reduced to 30–50 μL to accommodate lower DNA concentrations in the sample.

*Generation of barcodes:* The large nuclear subunit (28S) region of the ribosomal DNA repeat was first amplified with Rust2INV (Aime 2006)/LR6 or LR7 (Vilgalys and Hester 1990). Strong amplification products were sent for sequencing. Weak products were cleaned using ExoSap (ThermoFisher Science) with reactions of 7 μL consisting of 1 μL exonuclease, 1 μL shrimp alkaline phosphatase, and 5 μL of PCR DNA. 6 μL of cleaned template was then used in a nested PCR with primers Rust28SF (Aime et al. 2018)/LR5 or LR6 (Vilgalys and Hester 1990) following the protocols of Aime et al. (2018). When the ExoSap cleaning procedure did not provide ample purified DNA, the sodium acetate cleaning procedure was used (Openwetware.org). Sodium acetate was observed to produce higher quantities of purified DNA for use in the nested PCR protocol, but this protocol is labor-intensive. Samples were then sent to Genewiz for Sanger sequencing using the amplification primers. Returned forward and reverse reads were edited in Sequencher ver. 5.4 (Gene Codes Corp., Ann Arbor, Michigan, USA). Newly generated 28S sequences were BLAST searched against NCBI GenBank’s standard nr/nt nucleotide database to confirm the amplicon was a Pucciniales. Some isolates were sequenced twice to ensure the reproducibility and the accuracy of our methods.

*Confirmation of barcodes:* Each sequence from the same species were combined into a single Sequencher file and compared. All base reads were checked to ensure the accuracy of edits and to verify the species identification. Samples that did not BLAST as expected or did not match other replicates of the same species were verified with replicate sequencing and re-examination of the specimen(s).

*Database:* The Rust HUBB website (http://rustblast.aimelab.geddes.rcac.purdue.edu/) was built with SequenceServer software (2019) via Docker container. Sequencing data were verified and converted from FASTA to BLAST database files utilizing a separate virtual instance of SequenceServer before being uploaded and made available to Rust HUBB.

## RUST HUBB TOOL

RustHubb can be accessed directly at http://128.211.160.111/, or through an interface via the Aime Lab (https://www.aime-lab.com/) and Purdue University Herbaria (https://ag.purdue.edu/department/btny/herbaria/index.html) webpages.

**Fig. 3:**
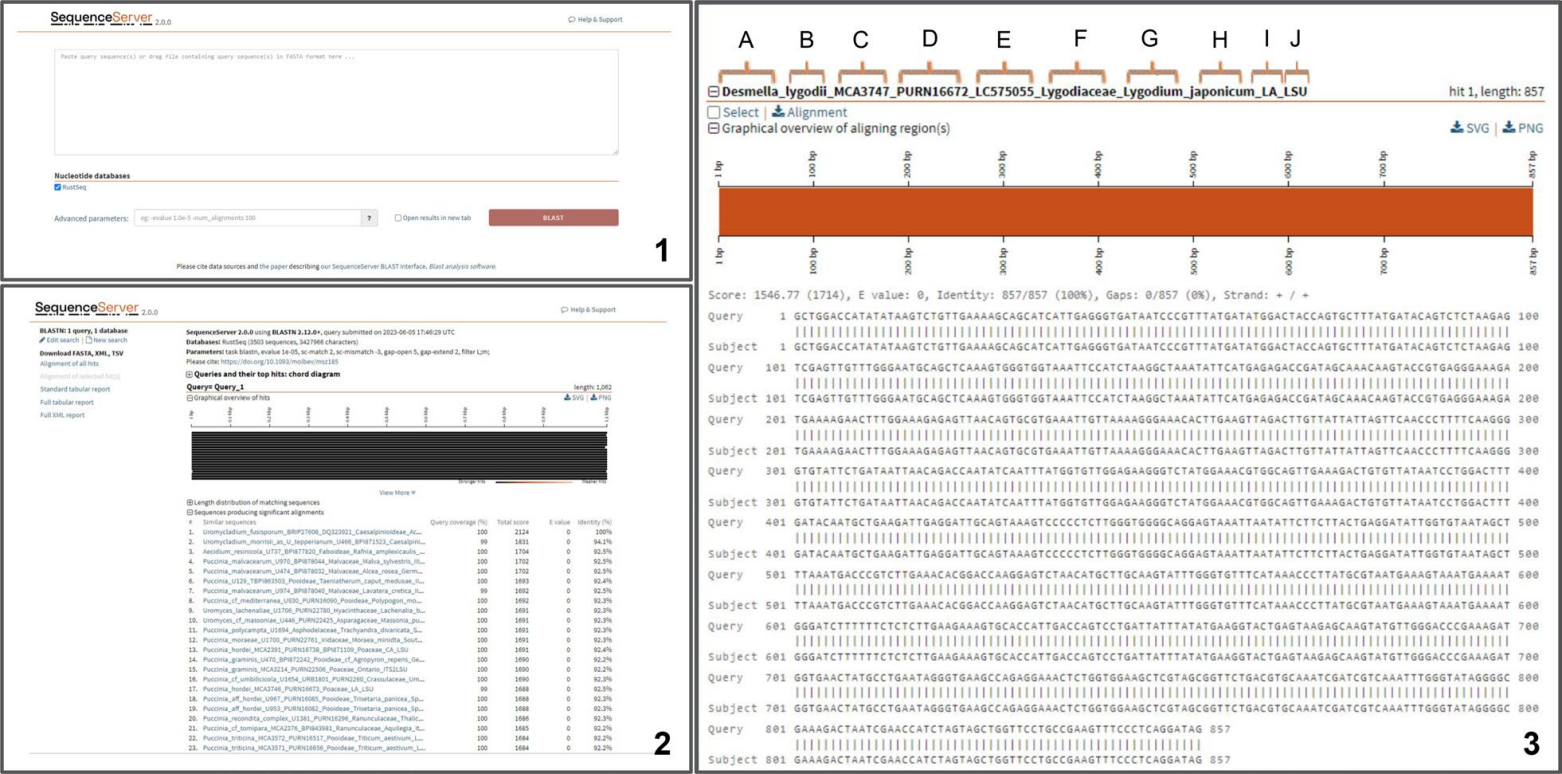
Rust HUBB interface. **1** Sequence Server entry page with paste block where users can input 28S sequences. **2** Search results will be returned in this format showing best matches. **3** Clicking on any match will give details including sequence length, base by base comparisons, and full header with sequence details as: A = Rust Genus; B = Rust species; C = collection number; D = Herbarium voucher number; E = GenBank accession number; F = Host family (or host subfamily for Poaceae, Asteraceae, and Fabaceae); G = Host genus; H = Host species; I = Country (or, for the US, state); J = Locus (where LSU = 28S D1-D3 region; ITS2LSU = LSU plus the preceding ITS2 region; D1-D2 = the D1-D2 region of the 28S only)

To search, input 28S barcodes of unknown rusts samples in the search bar (Fig. 3a). Results will be returned sorted by best match. As with BLAST searches, Query Coverage (%), Total Score, E Value, and Identity (%) metrics accompany each result (Fig. 3b). Corresponding data, such as collection and voucher numbers, GenBank number, host family, host genus and species, and country of origin of matches will be returned as displayed in Fig. 3c.

## RESULTS

Currently, our database contains verified data from 3771 sequences from 3734 specimens representing approximately 120 genera and 1150 species of Pucciniales, or ca. 15% of the known species diversity. Sequences are vouchered by herbarium specimens and names are updated regularly to reflect current taxonomic knowledge. Efforts to expand this resource to include data from most of the world’s known rust fungal species is ongoing.

## Discussion

It has been estimated that at least 20% of sequences in public repositories, such as GenBank, are incorrectly identified (Nilsson et al. 2006) which can be compounded by editing errors, and incorrectly applied names. Accurate identification of rust fungi is particularly exacerbated by the need for teliospores and incomplete data on host ranges even for well-known species. Rust HUBB is designed to facilitate fast and accurate identifications of unknown rust fungi from 28S DNA barcodes, regardless of morph or host.

This work was made possible due to the extensive collection of rust fungi within the Arthur Fungarium (PUR) at Purdue University. PUR was established in 1887 by Dr. Joseph C. Arthur, a highly accomplished researcher in mycology and plant pathology (McCain and Hennen 1990; Cummins 1978). Arthur described 309 species and 29 genera of rust fungi and published 12 volumes of the *North American Flora* (Arthur 1907–1931), as well as the only monograph of rust fungi of North America (Arthur 1934). George B. Cummins, who replaced Arthur (1938 to 1971) was equally prolific, generating a significant body of work including *The Illustrated Genera of Rust Fungi,* which is considered to be the “bible” of rust fungi (Cummins and Hiratsuka 2003). Significant Uredinologists that have worked at PUR include: Frank D. Kern, Joe F. Hennen, and John W. McCain, among many others. The collection contains over 168,000 specimens collected over several hundred years and from all around the world including ca. 4,500 type specimens. Projects such as this demonstrate the continued value of biological collections in modern scientific applications.

## Conclusion

Rust fungi are complex plant pathogens of great interest and concern to ecosystem health. With our publicly accessible, BLAST searchable database Rust HUBB, we hope to facilitate accurate and rapid identification of these fungi. We welcome contributions from the community; inquiries may be addressed to the corresponding author.

## Data Availability

The datasets and repository generated for this work are available in the Rust HUBB repository, http://128.211.160.111/.

## Abbreviations

Rust HUBB: Rust herbarium-based universal barcode BLAST
BLAST: Basic local alignment search tool
